# Having more virtual interaction partners during COVID-19 physical distancing measures may benefit mental health

**DOI:** 10.1038/s41598-021-97421-1

**Published:** 2021-09-14

**Authors:** Razia S. Sahi, Miriam E. Schwyck, Carolyn Parkinson, Naomi I. Eisenberger

**Affiliations:** 1 Department of Psychology, University of California Los Angeles, Los Angeles, CA 90095 USA; 2 Brain Research Institute, University of California Los Angeles, Los Angeles, CA 90095 USA

**Keywords:** Psychology, Human behaviour

## Abstract

Social interactions play an extremely important role in maintaining health and well-being. The COVID-19 pandemic and associated physical distancing measures, however, restricted the number of people one could physically interact with on a regular basis. A large percentage of social interactions moved online, resulting in reports of “Zoom fatigue,” or exhaustion from virtual interactions. These reports focused on how online communication differs from in-person communication, but it is possible that when in-person interactions are restricted, virtual interactions may benefit mental health overall. In a survey conducted near the beginning of the COVID-19 pandemic (*N*_2020_ = 230), we found that having a greater number of virtual interaction partners was associated with *better* mental health. This relationship was statistically mediated by decreased loneliness and increased perceptions of social support. We replicated these findings during the pandemic 1 year later (*N*_2021_ = 256) and found that these effects held even after controlling for the amount of time people spent interacting online. Convergent with previous literature on social interactions, these findings suggest that virtual interactions may benefit overall mental health, particularly during physical distancing and other circumstances where opportunities to interact in-person with different people are limited.

Open Science Framework repository: https://osf.io/6jsr2/.

## Introduction

A robust body of research demonstrates that social interactions are crucial in maintaining health and well-being^1,2^. Social interactions can mitigate the harmful effects of stress^3–5^, and provide protective benefits against a range of negative health outcomes. For example, social interactions can buffer against cognitive decline (associated with aging)^6^, dementia^6^, upper respiratory infections^7^, cancer recurrence^8^, and mortality^9–11^.

While social interactions likely shape health and well-being in a number of ways, one important outcome of social connection is reduced feelings of loneliness (i.e., subjective feelings of lacking companionship), which have consistently been linked to worse physical and mental health outcomes^12–15^. Relatedly, social interactions can also increase perceptions of social support, which can enable better emotion regulation and facilitate positive feelings of belonging and purpose^1,16,17^. Thus, social interactions can improve well-being by decreasing the harmful impact of negative emotional experiences, such as loneliness, while also increasing perceptions of social support and associated positive emotions that benefit overall mental health.

The COVID-19 pandemic and consequent physical distancing measures, however, restricted the number of people that individuals could interact with in-person on a regular basis. In other words, people have been more physically isolated than before the pandemic. Outside of the COVID-19 context, people often engage in a variety of in-person interactions in their everyday lives, including, those with a romantic partner, close family members, friends, and other members of one’s community^16^. In-person interactions that used to take place in passing, such as casual chats with neighbors or coworkers, became limited by governmental recommendations to work from home and maintain physical distance from others, reducing the number of in-person interaction partners that people could have on a regular basis.

Many social interactions moved online, including professional and recreational gatherings. This shift in how individuals interact with others on a regular basis has resulted in reports of “Zoom fatigue”, or exhaustion from virtual interactions. Countless news articles have reported on this phenomenon, describing the ways in which online interactions can increase mental load, including, but not limited to, less privacy when video-calling from living spaces, added stress from worrying about lighting, sound quality, and internet speed during important conversations, and minimal separation of different aspects of one’s life, such as work, friends, and family^18–21^. Given the recency of this drastic shift, limited research has examined how virtual interactions affect well-being. Empirical research comparing in-person and virtual interactions has begun to emerge suggesting that Zoom fatigue may arise from several nonverbal mechanisms that are unique to video communication (e.g., mirror anxiety, being physically trapped, “hypergaze” from a grid of staring faces, and increased cognitive load associated with producing and interpreting nonverbal communication in a virtual environment)^22,23^. These reports suggest that virtual interactions can be more taxing than in-person interactions. However, it remains unclear whether virtual interactions can benefit mental health in general, particularly when in-person social interactions, which have been repeatedly demonstrated to be important for health and well-being, are forcibly limited.

Given this unique historical moment and its potential to shape future work environments and social communication, the present research examined the relationship between virtual interactions and overall mental health. We focused our investigation on how the quantity of one’s virtual interaction partners (i.e., the number of people with whom individuals interact virtually on a regular basis) affects mental health. In a survey following the instantiation of COVID-19-related physical distancing measures in early-mid 2020 (*N*_2020_ = 230), including recommendations to work from home, wear a face covering in any public space, and avoid contact with individuals outside of one’s immediate household (i.e., maintain at least six feet of distance, severely limit time spent indoors with others), we tested two competing hypotheses: (1) based on prior literature on the benefits of social interactions, the number of one’s virtual interaction partners will be associated with better mental health; or (2) based on reports of increased fatigue associated with virtual interactions, the number of one’s virtual interaction partners will be associated with worse mental health. In line with prior literature, we followed-up on these analyses to explore potential mediators of the relationship between the quantity of one’s virtual interaction partners and mental health, including reduced loneliness and increased perceived social support.

We then replicated this study approximately 1 year later in early-mid 2021 (*N*_2021_ = 256) as the COVID-19 pandemic continued to unfold globally, but vaccinations became available in the United States (where this research was conducted) and governmental recommendations began shifting to allow physical contact between vaccinated individuals. During this time, much of education and work continued to take place remotely, and our undergraduate sample had just begun to get access to vaccinations, such that many everyday social interactions remained online rather than in-person. We were also interested in assessing the effects of the amount of time spent interacting with others online because of the emergence of preliminary research demonstrating that frequency of virtual interaction was not associated with well-being^24^ and that virtual interactions were more fatiguing and less enjoyable than in-person interactions^25^. In this second survey, we aimed to assess: (1) whether the positive association between the number of one’s virtual interaction partners and mental health was consistent between 2020 and 2021, even as the number of one’s in-person interactions were likely to have grown; and (2) the association between amount of time spent interacting with others online and mental health.

Because of the cross-sectional nature of our data, it is important to underscore that we cannot infer causality between our variables of interest and that our mediation analyses were primarily conducted to indicate possible mechanisms to pursue in future experimental or longitudinal research. However, it is nonetheless of critical importance to assess general trends in how changing social interactions are possibly shaping mental health in the real world during an unprecedented moment in history, that has the potential to permanently shift the way that we interact with others in the workplace and in everyday life. Thus, we aimed to shed light specifically on associations between virtual interactions (i.e., number of interaction partners and time spent interacting at both the daily and weekly level) and overall mental health at two time points during the COVID-19 pandemic.

## Methods

### Ethics statement

Our procedure was approved by the UCLA Institutional Review Board committee. All experiments were performed in accordance with the relevant guidelines and regulations. Informed consent was obtained from all participants.

### Sample size

The rationale for our target sample size of 300 participants for each study is based on the maximum number of participants that can be recruited through our institution’s subject pool in 1 year. For the first study, we began data collection soon after physical distancing measures went into place around the nation (dates of participation: April 27th, 2020–July 6th, 2020), and we aimed to enroll as many participants as possible in a small timeframe to capture the unique historical moment. One year later, we collected data in a new sample (dates of participation: April 17th, 2021–May 24th, 2021) as the pandemic continued to unfold globally, but when physical distancing measures were starting to ease due to vaccination rates in the United States, where this research was conducted.

### Participants

Participants for both studies were recruited from the UCLA Psychology Department’s undergraduate population using an online human participant management platform (SONA systems). Participants were granted university credit upon completion.

In the 2020 sample, the mean age of the final sample (*N* = 230) was 20.54 years (*SD* = 2.43). Approximately 88% of participants resided in California during participation and 3% lived outside of the U.S. Approximately 89% of participants reported living in cities with mandatory orders to work from home and maintain physical distance from others. Approximately 72% of participants were female, 26% male, < 1% non-binary, 34% Asian, 30% White, 13% Latinx, and 3% Black. The remaining participants selected another identity or chose not to answer.

In the 2021 sample, the mean age of the final sample (*N* = 256) was 20.56 years (*SD* = 3.11). Approximately 91% of participants resided in California during participation and 5% lived outside of the U.S. Approximately 42% of participants reported living in cities with mandatory orders to work from home and maintain physical distance from others. Approximately 76% of participants were female, 22% male, 1% non-binary, 38% Asian, 24% White, 12% Latinx, and 3% Black. The remaining participants selected another identity or chose not to answer.

### Measures

To assess the extent to which social interactions impacted mental health during physical distancing, we used the following measures: (i) the short form of the Mental Health Continuum (MHC-SF)^26^ measures positive mental health (i.e., emotional, psychological, and social well-being) with a total of 14 items such as: “During the past month, how often did you feel happy/good at managing the responsibilities of your daily life/that your life has a sense of direction or meaning to it?”; and (ii) self-reported average number of daily and weekly virtual interaction partners (i.e., “Currently, how many different people do you actively interact with [daily/weekly]?”). For our measure of virtual interaction, we asked participants to focus on meetings over Zoom, phone calls, texts, virtual gaming, or other similar means of communication that did not involve in-person interaction. While our primary research questions centered on the effects of how many different virtual interaction partners participants had, we simultaneously measured participants’ self-reported average number of daily and weekly in-person interaction partners. For these items, we asked participants to focus on people they socialized with face-to-face. In 2021, we additionally included a measure of time spent interacting with others, separately for virtual and in-person interactions, at both the daily and weekly level (i.e., “Currently, how many hours do you actively interact with people [in-person/virtually; daily/weekly]?”).

To assess whether loneliness and perceived social support mediated the relationship between number of virtual interaction partners and mental health, we used the following measures: (i) UCLA Loneliness Scale^27^, which includes a total of 20 items such as: “How often do you feel alone/that no one really knows you well/that there are people you can turn to?”; and (ii) the Social Provisions Scale^28^, which includes a total of 24 items such as: “There is someone I could talk to about important decisions in my life,” “There are people who enjoy the same social activities I do,” and “I have relationships where my competence and skill are recognized.” Detailed information for our measures, including access to the full list of items for each scale, are provided in our Open Science Framework (OSF) repository.

### Procedure

This data was collected as part of a larger multi-investigator project examining individuals’ social interactions and experiences during the COVID-19 pandemic. A full list of measures included in the parent project is available upon request. Participants signed up for the study through our institution’s subject pool where they could access a link to our survey hosted on Qualtrics.com. The 2020 survey was estimated to take approximately 50 min to complete, and the 2021 survey was estimated to take approximately 30 min to complete.

### Exclusion criteria

Participants who reported being younger than 18 or not being proficient in English were not enrolled in the study. Since these studies were completed online (*N*_2020_ = 292; *N*_2021_ = 300), we used several criteria to clean our dataset. Specifically, people were excluded for completing the survey in less than half the anticipated study length (i.e., < 25 min in 2020, < 15 min in 2021; *N*_2020_ = 20; *N*_2021_ = 3), for failing attention checks (*N*_2020_ = 31; *N*_2021_ = 25), and for being outliers (more than three standard deviations away from the mean) on at least one of the primary variables of interest *N*_2020_ = 11; *N*_2021_ = 12). Additionally, in the 2021 sample, participants were excluded from the final sample if they reported having completed the same survey in 2020 (*N*_2021_ = 4). Please see Table S1 for demographic information of the excluded participants, which suggest that included and excluded participants approximately matched in terms of demographic characteristics.

### Data normality and transformations

All variables of interest were visually examined for normal distributions. The four variables measuring number of interaction partners (i.e., number of daily/weekly virtual/in-person interaction partners) in both studies were skewed right. In 2021, our measures of time spent interacting with others were also skewed right. As such, these variables were each log-transformed before further analysis.

### Analyses

All analyses were conducted using the statistical software R (Version 3.6.1)^29^. Our data and analysis materials are hosted on OSF. First, we tested the effect of the number of participants’ virtual interaction partners on their mental health during the COVID-19 pandemic by running simple regression models including either number of daily virtual interaction partners or number of weekly virtual interaction partners as the predictor of mental health. We followed up on these analyses with simple mediation analyses to examine whether loneliness (i.e., UCLA Loneliness Scale) and perceived social support (i.e., Social Provisions Scale) statistically mediated the relationship between the number of one’s daily/weekly virtual interaction partners and mental health. We examined these mediators separately (rather than creating a composite variable or in a parallel mediation model) since prior literature has separately examined the mediating effects of these two variables on the relationship between social interactions and mental health, and our measures of loneliness and perceived support were highly correlated (*r* = − 0.78), which can create issues of multicollinearity. Using the “mediation” package in R^30,31^, we estimated average causal mediation effects (ACMEs) and average direct effects (ADEs), and tested the significance of these effects using bootstrapping procedures. Unstandardized indirect effects (i.e., ACMEs) were computed for each of 10,000 bootstrapped samples, and the 95% confidence interval was computed by determining the indirect effects at the 2.5th and 97.5th percentiles.

We would like to note that although our model is described as a statistical mediational model in which each examined variable is separated in time, we are not suggesting a time-lagged mediational model here (e.g., number of virtual interaction partners at Time 1 will alter loneliness at Time 2, which will then alter mental health at Time 3). Rather, we assessed whether decreased loneliness and increased perceptions of social support *underlie* associations between virtual interaction and overall mental health. Although we recognize that mediational analyses have the connotation of being time-lagged, our proposed model fits with the standard definition of statistical mediation, which occurs when one variable (in this case, loneliness or perceived social support) helps to explain the association between a predictor of interest (i.e., number of virtual interaction partners) and a particular outcome (i.e., overall mental health). Thus, while we will use the same formal tests of mediation, these analyses should be conceptualized as a ‘levels of analysis’ mediation (i.e., do decreased loneliness and increased perceived social support underlie positive associations between virtual interactions and mental health?) rather than a ‘time-lagged’ mediation (i.e., do decreased loneliness and increased perceived social support improve mental health at a later timepoint?).

We performed the same analytic procedure in both the 2020 and 2021 datasets. Overall mental health did not significantly change from 2020 (*M* = 37.56, *SD* = 14.73) to 2021 (*M* = 39.60, *SD* = 13.52), *t*(459.04) = 1.57, *p* = 0.117. Since the 2021 study included a measure of time spent interacting with others, we additionally tested whether time spent interacting with others online was associated with mental health.

The primary focus of our inquiry was the relationship between virtual interactions and mental health during the COVID-19 pandemic. However, because we also assessed the number of peoples’ in-person interaction partners in both datasets, and time spent interacting with others in-person in the 2021 dataset, we also examined: (i) the relationship between the number of one’s in-person interaction partners and mental health; (ii) whether this relationship was statistically mediated by decreased loneliness and increased perceived social support; and (iii) the relationship between time spent interacting with others in-person and mental health. These analyses and results are described in our Supplemental Materials (S3).

## Results

### Is the quantity of one’s virtual interaction partners associated with better or worse mental health?

#### 2020

**Figure 1:**
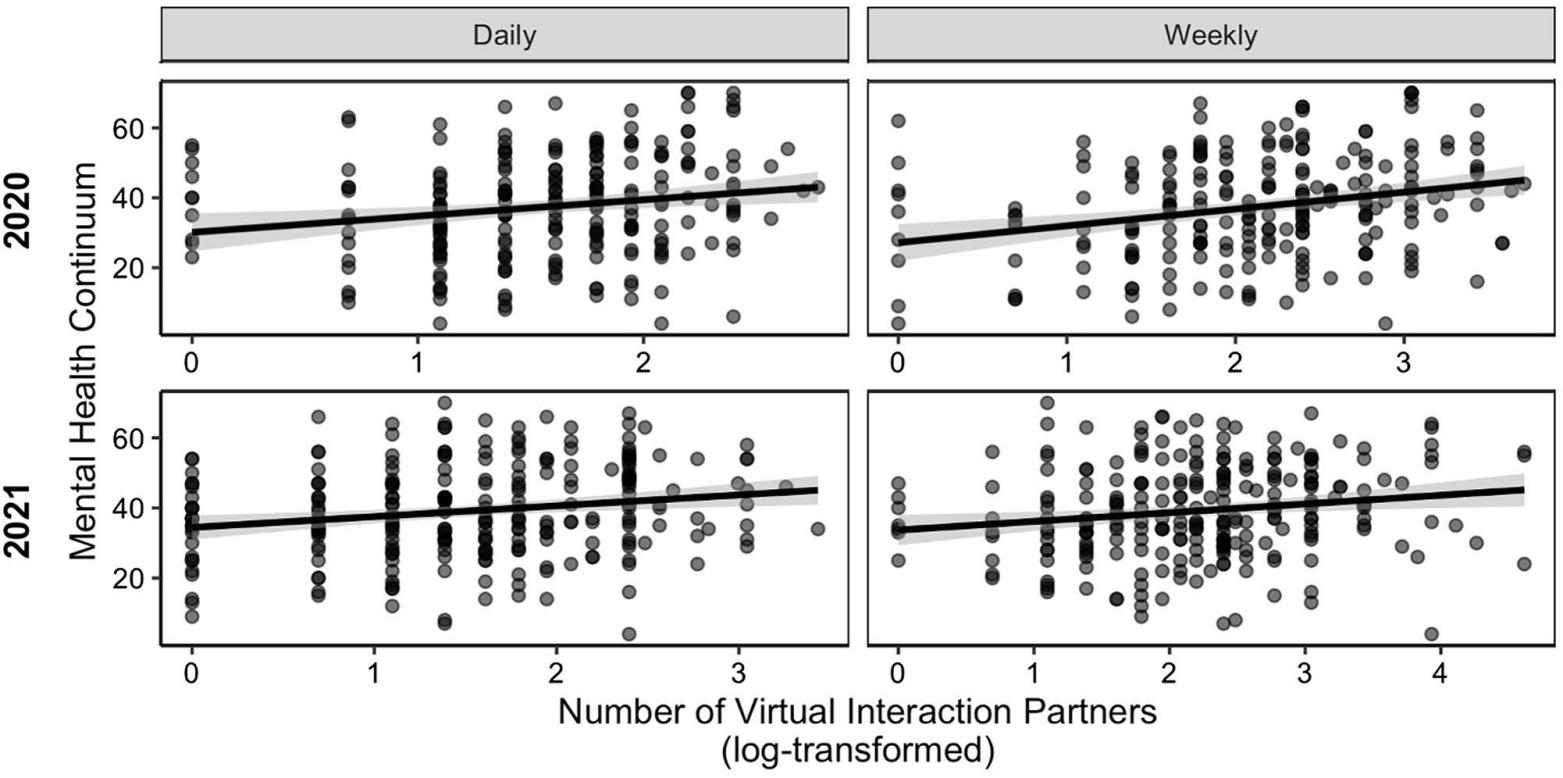
Association between number of virtual interaction partners and mental health. Mental health, as measured by the Mental Health Continuum, was positively associated with the number of people participants virtually interacted with on a daily and weekly basis in both 2020 and 2021. 95% confidence intervals around the linear regression line are shown^29^.

Congruent with past literature on the benefits of social interactions, we found that the quantity of one’s virtual interaction partners was associated with better mental health at both the daily level, β = 4.68, 95% CI [1.46, 7.90], *t*(224) = 2.86, *p* = 0.005, and the weekly level, β = 4.83, 95% CI [2.53, 7.12], *t*(224) = 4.14, *p* < 0.001 (Fig. 1).

#### 2021

Consistent with our 2020 findings, the quantity of one’s virtual interaction partners was associated with better mental health during physical distancing in 2021 at both the daily level, β = 3.23, 95% CI [1.19, 5.27], *t*(251) = 3.11, *p* = 0.002, and the weekly level, β = 2.61, 95% CI [0.76, 4.46], *t*(251) = 2.78, *p* = 0.006 (Fig. 1).

#### Change from 2020 to 2021

There was a marginally significant increase in average number of daily virtual interaction partners between 2020 and 2021 (Table S2), *t*(418.63) = 1.83, *p* = 0.069, and a significant increase in number of weekly virtual interaction partners from 2020 to 2021, *t*(397.51) = 2.32, *p* = 0.021.

### Is the relationship between the number of one’s virtual interaction partners and mental health mediated by decreased loneliness?

#### 2020

**Figure 2:**
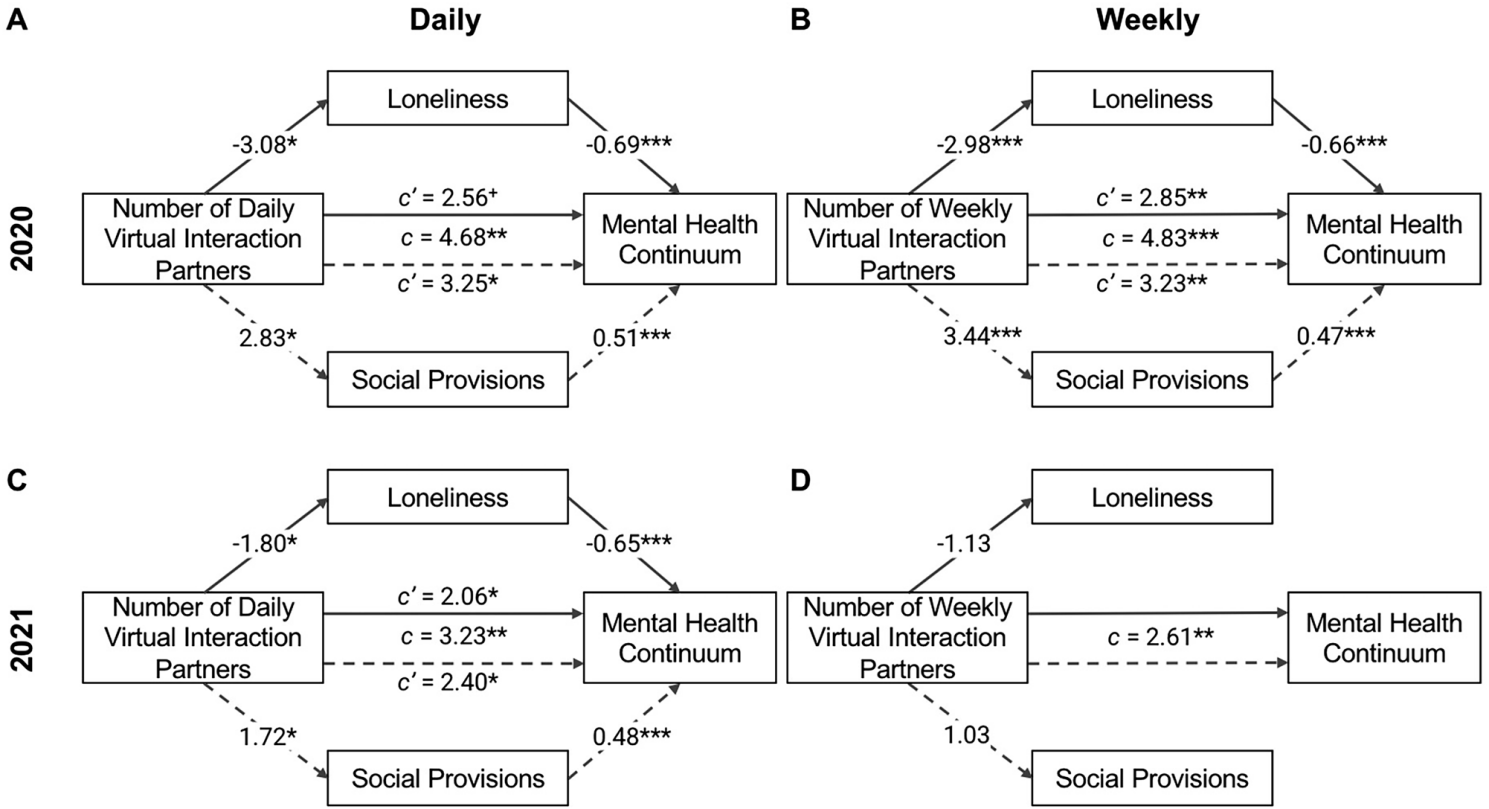
Reduced loneliness and greater perceived support mediate the positive relationship between number of virtual interaction partners and mental health. Simple mediation analyses were performed on the positive relationship between mental health and number of interaction partners. Regression coefficients and associated significance levels for these analyses are shown. The relationship between interaction partners and mental health was statistically mediated by reduced loneliness and increased perceived social support in all models, except for the relationship between mental health and weekly interaction partners in 2021. Note that number of weekly interaction partners in 2021 was not predictive of either loneliness or social provisions, so no further mediation analyses were performed. *p < 0.050, **p < 0.010, ***p < 0.001.

To test if decreased loneliness helped to explain the relationship between number of virtual interaction partners and mental health, we ran statistical mediation analyses. Quantity of daily virtual interaction partners was negatively associated with loneliness, β = − 3.08, 95% CI [− 5.42, − 0.74], *t*(224) = − 2.59, *p* = 0.010. When controlling for the effect of quantity of daily virtual interaction partners on mental health, β = 2.56, 95% CI [− 0.28, 5.39], *t*(223) = 1.78, *p* = 0.077, loneliness was negatively associated with mental health, β = -0.69, 95% CI [− 0.85, − 0.53], *t*(223) = − 8.65, *p* < 0.001. There was a significant indirect effect (i.e., mediation effect) of daily virtual interaction partners on mental health, β_ACME_ = 2.12, 95% CI [0.56, 3.99], *p* = 0.006, and a marginally significant direct effect of daily virtual interaction partners on mental health (i.e., effect of interaction partners on mental health independent of loneliness), β_ADE_ = 2.56, 95% CI [− 0.33, 5.71], *p* = 0.085. Thus, the effect of one’s number of daily virtual interaction partners on mental health was fully mediated by decreased loneliness in 2020 (Fig. 2).

Similar to the effects of daily virtual interaction partners described above, quantity of weekly virtual interaction partners was negatively associated with loneliness, β = − 2.98, 95% CI [− 4.66, − 1.30], *t*(224) = − 3.49, *p* < 0.001, and when controlling for the effect of quantity of weekly virtual interaction partners on mental health, β = 2.85, 95% CI [0.78, 4.91], *t*(223) = 2.72, *p* = 0.007, loneliness was negatively associated with mental health, β = − 0.66, 95% CI [− 0.82, − 0.51], *t*(223) = − 8.32, *p* < 0.001. There was a significant indirect effect, β_ACME_ = 1.98, 95% CI [0.81, 3.35], *p* = 0.001, and significant direct effect, β_ADE_ = 2.85, 95% CI [0.79, 5.01], *p* = 0.007, of weekly virtual interaction partners on mental health, indicating that the effect of number of weekly virtual interaction partners on mental health was partially mediated by decreased loneliness in 2020.

#### 2021

Consistent with our findings from 2020, quantity of daily virtual interaction partners was negatively associated with loneliness, β = − 1.80, 95% CI [− 3.33, − 0.26], *t*(251) = − 2.30, *p* = 0.022. When controlling for quantity of daily virtual interaction partners, β = 2.06, 95% CI [0.26, 3.87], *t*(250) = 2.25, *p* = 0.025, loneliness was negatively associated with mental health, β = − 0.65, 95% CI [− 0.79, − 0.50], *t*(250) = − 8.85, *p* < 0.001. There was a significant indirect effect of daily virtual interaction partners on mental health, β_ACME_ = 1.17, 95% CI [0.08, 2.30], *p* = 0.033, and a significant direct effect of daily virtual interaction partners on mental health, β_ADE_ = 2.06, 95% CI [0.37, 3.78], *p* = 0.018. Thus, the effect of one’s number of daily virtual interaction partners on mental health was partially mediated by decreased loneliness in 2021.

In 2021, there was no significant association between number of weekly virtual interaction partners and loneliness, β = − 1.13, 95% CI [− 2.52, 0.27], *t*(251) = − 1.59, *p* = 0.113. Thus, no further mediation analyses were performed.

### Is the relationship between the number of one’s virtual interaction partners and mental health mediated by greater perceived social support?

#### 2020

Next, we tested if perceived social support also mediated the relationship between virtual interaction partners and mental health. Quantity of daily virtual interactions was positively associated with perceived social support, β = 2.83, 95% CI [0.63, 5.02], *t*(224) = 2.53, *p* = 0.012. When controlling for quantity of daily virtual interaction partners, β = 3.25, 95% CI [0.18, 6.32], *t*(223) = 2.08, *p* = 0.038, perceived support was positively associated with mental health, β = 0.51, 95% CI [0.33, 0.69], *t*(223) = 5.51, *p* < 0.001. Thus, the effect of one’s number of daily virtual interaction partners on mental health was partially mediated by increased perceived support in 2020, β_ACME_ = 1.43, 95% CI [0.32, 2.88], *p* = 0.009, β_ADE_ = 3.25, 95% CI [0.16, 6.57], *p* = 0.039 (Fig. 2).

Quantity of weekly virtual interaction partners was positively associated with perceived social support, β = 3.44, 95% CI [1.88, 4.99], *t*(224) = 4.35, *p* < 0.001, and when controlling for quantity of weekly virtual interaction partners, β = 3.23, 95% CI [0.95, 5.50], *t*(223) = 2.80, *p* = 0.006, perceived support was positively associated with mental health, β = 0.47, 95% CI [0.28, 0.65], *t*(223) = 4.96, *p* < 0.001. The effect of number of weekly virtual interaction partners on mental health was partially mediated by increased perceived support in 2020, β_ACME_ = 1.60, 95% CI [0.64, 2.85], *p* < 0.001, β_ADE_ = 3.23, 95% CI [0.83, 5.70], *p* = 0.009.

#### 2021

Consistent with our findings in 2020, quantity of daily virtual interactions was positively associated with perceived social support in 2021, β = 1.72, 95% CI [0.13, 3.31], *t*(251) = 2.13, *p* = 0.034. When controlling for quantity of daily virtual interaction partners, β = 2.40, 95% CI [0.49, 4.31], *t*(250) = 2.47, *p* = 0.014, perceived support was positively associated with mental health, β = 0.48, 95% CI [0.33, 0.63], *t*(250) = 6.41, *p* < 0.001. The effect of one’s number of daily virtual interaction partners on mental health was partially mediated by increased perceived support in 2021, β_ACME_ = 0.83, 95% CI [0.02, 1.80], *p* = 0.045, β_ADE_ = 2.40, 95% CI [0.55, 4.21], *p* = 0.010 (Fig. 2).

In 2021, there was no significant association between number of weekly virtual interaction partners and perceived social support, β = 1.03, 95% CI [− 0.41, 2.47], *t*(251) = 1.41, *p* = 0.161. As such, no further mediation analyses were performed.

### Is time spent interacting with others online associated with mental health?

Thus far we have discussed the effects of interacting with different people on mental health. It may be, however, that time spent interacting with others online contributes to Zoom fatigue, which could worsen overall mental health. While we did not have a measure of time spent interacting with others in the 2020 survey, in the 2021 survey we asked participants how many hours they spent virtually interacting with others on a daily and weekly basis. We found no relationship between daily virtual interaction hours and mental health, β = 1.86, 95% CI [− 0.94, 4.57], *t*(251) = 1.30, *p* = 0.195, but we did find a significant positive relationship between weekly virtual interaction hours and mental health, β = 1.86, 95% CI [0.12, 3.61], *t*(251) = 2.10, *p* = 0.037.

To further understand the relationship between virtual interaction and mental health, we tested whether the number of one’s virtual interaction partners predicted mental health over and above time spent interacting online by including both number of virtual interaction partners and time spent interacting online in linear regression models, separately for daily and weekly interactions. When controlling for time spent interacting online, β = − 0.22, 95% CI [− 3.28, 2.85], *t*(250) = − 0.14, *p* = 0.890, we found that number of virtual interaction partners was positively associated with mental health at the daily level, β = 3.30, 95% CI [1.00, 5.61], *t*(250) = 2.82, *p* = 0.005. Similarly, when controlling for time spent interacting online, β = 0.93, 95% CI [− 1.02, 2.89], *t*(250) = 0.94, *p* = 0.349, number of virtual interaction partners was positively associated with mental health at the weekly level, β = 2.16, 95% CI [0.07, 4.24], *t*(250) = 2.04, *p* = 0.043.

## Discussion

The present study examined the relationship between the number of one’s virtual interaction partners during COVID-19 physical distancing and overall mental health. In a survey conducted in early-mid 2020, we found that the more daily and weekly virtual interaction partners one had, the better their mental health. This relationship was mediated by decreased loneliness and increased perceptions of social support. We replicated these effects in a second sample collected in early-mid 2021, and additionally found that the amount of time spent interacting with others online was *not* significantly associated with mental health at the daily level, in line with recent work suggesting that frequency of virtual interactions was not associated with well-being^24^. However, time spent interacting with others online was significantly positively associated with mental health at the weekly level, suggesting that time spent online may also benefit mental health.

These findings extend a robust body of work demonstrating that social interactions are important for health and well-being. Virtual interactions and their associated practical and social challenges (e.g., technical difficulties, minimization of non-verbal cues) have been a reported source of stress for many people throughout the pandemic^18–23^, but our research suggests that virtual interactions—and in particular, interacting with many different people online—may nonetheless confer mental health benefits during physical distancing when in-person interactions are limited. These findings are consistent with recent work suggesting that people consistently undervalue the overall benefit of voice-based interactions such as video chat and phone calls^32^.

Given that the COVID-19 pandemic has the potential to shape future work norms, with companies and universities increasingly experimenting with and adopting remote work models^33–35^, it is crucial to assess the impact of virtual interactions on our well-being. Interestingly, virtual interactions may benefit mental health through similar mechanisms as in-person interactions typically do insofar as virtual interactions afford the opportunity to expand the number of social interaction partners one can have, particularly while maintaining physical distance from others. Even as physical distancing measures change over time and we have more in-person access to the people around us, this work has implications for individuals with limited mobility, such as older individuals, for whom virtual interactions have been found to reduce feelings of loneliness and increase connections with friends and family^36^.

Although this research elucidates the possible protective benefits of virtual interactions, particularly as it relates to expanding the number of people one interacts with on a regular basis, preliminary research also suggests that virtual interactions are less enjoyable, more fatiguing, and more taxing in general than in-person interactions^22,25^. It is possible that this discrepancy between in-person and virtual interactions will shrink as we become more accustomed to interacting virtually, and virtual interfaces become increasingly user-friendly and interactive. Interestingly, our data suggest that even as the average number of in-person interactions has expanded at the daily and weekly level from 2020 to 2021 in our sample, so have the weekly number of virtual interaction partners. Thus, future work can experimentally parse how specific features of virtual interactions (e.g., layout, interactive tools) and different methods of communicating virtually (e.g., video calls, phone calls, virtual games, etc.) shape well-being, mental health, and feelings of social connection in order to improve the quality of virtual interactions^22,32^. Importantly, some features of in-person interaction cannot be approximated in a virtual environment. For example, in-person interactions can facilitate affectionate touch, which has been robustly demonstrated to promote relational, psychological, and physical well-being across age groups and relationships^37,38^. Thus, virtual interactions may not adequately replace in-person interactions in the long-run, particularly if experiences like affectionate touch and shared sensory experiences are mechanistically important in buffering against the inverse effects of loneliness.

Importantly, our work is not meant to discount experiences of burnout associated with virtual interactions. A primary source of “Zoom fatigue” may be the cognitive effort expended to adjust to a new interactive context, as well as the physical toll of staying in one’s chair and looking at a bright screen for extended periods of time. Our research indicated that the number of one’s virtual interaction partners at the daily and weekly level was positively associated with overall mental health, and that time spent interacting with others online at the weekly level was also positively associated with overall mental health. Thus, it will be important for future work to continue examining sources of Zoom fatigue, and how to mitigate these effects without losing the potential benefits of virtual interaction.

A crucial limitation of our results is that they are derived from cross-sectional data, so we are not able to determine the directionality of the relationship between the number of one’s virtual interaction partners and mental health. In other words, it is possible that individuals with better overall mental health are more likely to virtually interact with a large number of people, as opposed to the other way around. Importantly, time spent interacting with others online was not associated with mental health at the daily level, and although it was positively associated with mental health at the weekly level, number of weekly interaction partners was positively associated with mental health when controlling for weekly time spent interacting online. These findings suggest that those who are more social in general (i.e., spend more time communicating with others) are not necessarily those with stronger mental health, but rather that there is something specific about the association between *the number of different people* one interacts with on a regular basis and mental health. Of course, future research using longitudinal or experimental approaches are imperative to further understand this association. Large-scale survey data can additionally be used to explore how factors such as occupational or social network status relate to social interaction during COVID-19. However, our research is among the first to test both number of interaction partners and time spent interacting with others virtually during COVID-19, and indicates that it is worth considering the potential mental health benefits of virtual interactions during COVID-19 and other contexts that restrict the number of people we can regularly interact with. Thus, this work lays the groundwork for future research to continue to examine the ways in which in-person versus virtual interactions shape well-being as our society adjusts to new ways of communicating in the workplace and everyday life.

In sum, this work answers an important question about the relationship between virtual interactions and mental health through these challenging times. While we may be tempted to limit our virtual interactions as much as possible given the fatigue associated with such forms of communication relative to in-person communication, this research suggests that it is worth maintaining a variety of interaction partners as we continue to navigate an unprecedented environment.

## Supplementary Information

Supplementary Information.

## Data Availability

All de-identified data and data analysis scripts for this manuscript have been made publicly available and are accessible at: https://osf.io/6jsr2/.
