# Supplementary material for: Having more virtual interaction partners during COVID-19 physical distancing measures may benefit mental health
Source: Sci Rep. 2021 Sep 14;11:18273. doi: 10.1038/s41598-021-97421-1 (PMC8440653; doi:10.1038/s41598-021-97421-1)
Supplement: Supplementary file 1 — Supplementary Information. [file 41598_2021_97421_MOESM1_ESM.pdf]

Supplementary Materials for:

Having more virtual interaction partners during COVID-19 physical distancing measures may benefit mental health

Razia S. Sahi, Miriam E. Weaverdyck, Carolyn Parkinson, & Naomi I. Eisenberger

S1. Comparison of Demographic Information of Included versus Excluded Participants

Table S1. Demographic Information of Participants

	**2020**		**2021**	
	**Included (*N* = 230)**	**Excluded (*N* = 66)**	**Included (*N* = 256)**	**Excluded (*N* = 44)**
**<u>Gender</u>**				
**Male**	26%	32%	22%	16%
**Female**	72%	64%	76%	80%
**Non-Binary**	<1%	2%	1%	0%
**<u>Ethnicity</u>**				
**Asian**	34%	35%	38%	36%
**White**	30%	39%	24%	18%
**Latinx**	13%	9%	12%	16%
**Black**	3%	2%	3%	5%

S2. Number of Virtual Interaction Partners and Hours Spent Interacting Virtually

Table S2. Average number of virtual interaction partners and hours spent interacting virtually during the COVID-19 pandemic at a daily and weekly level

	Number of Interaction Partners		Number of Interaction Hours
	2020	2021	2021
Daily	4.58 (2.99)**	5.26 (5.01)**	2.95 (2.51)
Weekly	10.18 (8.21)***	12.72 (15.10)**	16.01 (15.92)*

Note. Standard deviations are provided in parentheses. Asterisks indicate a significant relationship with overall mental health. *$p < .05$, **$p < .01$, ***$p < .001$

S3. The Effect of In-Person Interactions on Mental Health and Possible Mediators of this Relationship

The average number of in-person interaction partners that people had significantly increased from 2020 to 2021 at both the daily, $t(359.97) = -3.52$, $p < .001$, and weekly level, $t(376.54) = -5.70$, $p < .001$. In 2021, participants also reported how many hours they interact with others in-person at a daily and weekly level. Since the number of in-person interaction hours would likely be skewed by the number of people one lives with, we broke down this question even further, asking participants how much in-person time they spend with people they live with as well as with people they do not live with. See Table S2 for a summary of individuals' average in-person interactions in 2020 and 2021.

Table S3. Average number of in-person interaction partners and hours during the COVID-19 pandemic at a daily and weekly level

	Number of Interaction Partners		Number of Interaction Hours (2021)	
	2020	2021	Cohabitants	Non-cohabitants
Daily	3.12 (1.90)	4.15 (4.19)***	6.59 (5.44)	2.18 (2.85) $^{+}$
Weekly	5.38 (4.76)***	9.29 (9.65)**	41.08 (37.65) $^{+}$	13.19 (18.05) $^{+}$

Note. Standard deviations are provided in parentheses. Asterisks indicate a significant relationship with overall mental health. +$p < .1$, *$p < .05$, **$p < .01$, ***$p < .001$

Is the quantity of one's in-person interaction partners associated with better or worse mental health?

We first examined the relationship between the number of one's in-person interaction partners and their mental health during COVID-19 physical distancing measures. In 2020, we found a significant positive relationship between the number of one's weekly in-person interaction partners and their mental health, β = 4.79, 95% CI [2.31, 7.27], $t(224) = 3.80$, $p < .001$, but not between their daily number of in-person interactions and mental health, β = 3.03,

95% CI [-0.96, 7.01], $t(224) = 1.50$, $p = .136$. In 2021, we found significant positive relationships between the number of one's in-person interaction partners and their mental health at both the daily, $\beta = 4.99$, 95% CI [2.23, 7.74], $t(251) = 3.57$, $p < .001$, and weekly level, $\beta = 2.94$, 95% CI [0.88, 5.00], $t(251) = 2.81$, $p = .005$.

Is the relationship between the number of one's virtual interaction partners and mental health mediated by decreased loneliness or increased perceived social support?

2020. The number of one's weekly in-person interaction partners was negatively associated with loneliness, $\beta = -3.10$, 95% CI [-4.95, -1.25], $t(221) = -3.30$, $p = .001$. When controlling for quantity of weekly in-person interactions, $\beta = 2.77$, 95% CI [0.52, 5.02], $t(220) = 2.43$, $p = .016$, loneliness was negatively associated with mental health, $\beta = -0.70$, 95% CI [-0.85, -0.54], $t(220) = -8.73$, $p < .001$. The effect of number of one's weekly in-person interaction partners on mental health was partially mediated by decreased loneliness, $\beta_{ACME} = 2.16$, 95% CI [0.84, 3.71], $p < .001$, $\beta_{ADE} = 2.77$, 95% CI [0.47, 5.00], $p = .019$.

Additionally, number of weekly in-person interaction partners was positively associated with perceived social support, $\beta = 2.81$, 95% CI [1.08, 4.54], $t(221) = 3.19$, $p = .002$. When controlling for quantity of weekly in-person interaction partners, $\beta = 3.47$, 95% CI [1.03, 5.91], $t(220) = 2.81$, $p = .005$, perceived support was positively associated with mental health, $\beta = 0.52$, 95% CI [0.34, 0.70], $t(220) = 5.62$, $p < .001$. The effect of the number of one's weekly in-person interaction partners on mental health was partially mediated by increased perceived social support, $\beta_{ACME} = 1.46$, 95% CI [0.53, 2.65], $p = .001$, $\beta_{ADE} = 3.47$, 95% CI [1.07, 5.82], $p = .003$.

2021. We found that both loneliness and perceived social support fully mediated the relationship between number of interaction partners and mental health at both the daily and weekly level. Specifically, daily in-person interaction partners was negatively associated with

loneliness, $\beta = -4.59$, 95% CI [-6.62, -2.56], $t(251) = -4.45$, $p < .001$, and when controlling for number of daily in-person interaction partners, $\beta = 2.05$, 95% CI [-0.48, 4.58], $t(250) = 1.60$, $p = .111$, loneliness was negatively associated with mental health, $\beta = -0.64$, 95% CI [-0.79, -0.49], $t(250) = -8.45$, $p < .001$. The effect of the number of one's daily in-person interaction partners on mental health was fully mediated by decreased loneliness, $\beta_{ACME} = 2.94$, 95% CI [1.59, 4.34], $p < .001$, $\beta_{ADE} = 2.05$, 95% CI [-0.75, 4.70], $p = .145$.

Number of weekly in-person interaction partners was negatively associated with loneliness, $\beta = -2.55$, 95% CI [-4.08, -1.03], $t(251) = -3.29$, $p = .001$, and when controlling for number of weekly in-person interaction partners, $\beta = 1.27$, 95% CI [-0.57, 3.12], $t(250) = 1.36$, $p = .175$, loneliness was negatively associated with mental health, $\beta = -0.65$, 95% CI [-0.80, -0.51], $t(250) = -8.75$, $p < .001$. The effect of the number of one's weekly in-person interaction partners on mental health was fully mediated by decreased loneliness, $\beta_{ACME} = 1.67$, 95% CI [0.60, 2.69], $p = .003$, $\beta_{ADE} = 1.27$, 95% CI [-0.71, 3.27], $p = .200$.

Number of daily in-person interaction partners was positively associated with perceived social support, $\beta = 5.47$, 95% CI [3.40, 7.54], $t(251) = 5.21$, $p < .001$, and when controlling for number of daily in-person interaction partners, $\beta = 2.45$, 95% CI [-0.27, 5.18], $t(250) = 1.77$, $p = .077$, perceived social support was positively associated with mental health, $\beta = 0.46$, 95% CI [0.31, 0.62], $t(250) = 5.87$, $p < .001$. The effect of the number of one's daily in-person interaction partners on mental health was fully mediated by increased perceptions of social support, $\beta_{ACME} = 2.54$, 95% CI [1.32, 3.98], $p < .001$, $\beta_{ADE} = 2.45$, 95% CI [-0.68, 5.39], $p = .129$.

Number of weekly in-person interaction partners was positively associated with perceived support, $\beta = 3.33$, 95% CI [1.77, 4.89], $t(251) = 4.21$, $p < .001$, and when controlling for number of weekly in-person interaction partners, $\beta = 1.34$, 95% CI [-0.65, 3.33], $t(250) =$

1.32, $p = .187$, perceived social support was positively associated with mental health, $\beta = 0.48$, 95% CI [0.33, 0.63], $t(250) = 6.18$, $p < .001$. The effect of the number of one's weekly in-person interaction partners on mental health was fully mediated by decreased loneliness, $\beta_{ACME} = 1.60$, 95% CI [0.69, 2.59], $p < .001$, $\beta_{ADE} = 1.234$, 95% CI [-0.85, 3.51], $p = .229$.

Is time spent interacting with others online associated with mental health?

It could be that the amount of time that one spends with others, rather than the number of different people one interacts with, relates to overall mental health. To test this, we ran linear models with mental health as the outcome variable, and the number of hours one spends with people they live with and the number of hours one spends with the people they do not live with as the two predictors. We found that mental health was not significantly associated with the daily number of hours one interacts with their cohabitants, $\beta = 0.76$, 95% CI [-1.38, 2.89], $t(250) = 0.70$, $p = .487$, and it was only marginally associated with the daily number of hours spent interacting with people outside of one's household, $\beta = 1.96$, 95% CI [-0.22, 4.14], $t(250) = 1.77$, $p = .078$. Both predictors were marginally predictive at the weekly level, $\beta_{cohabitant} = 1.18$, 95% CI [-0.20, 2.57], $t(250) = 1.68$, $p = .094$; $\beta_{non\text{-}cohabitant} = 1.27$, 95% CI [-0.14, 2.69], $t(250) = 1.77$, $p = .078$.

Next, we tested if the number of one's in-person interaction partners predicted mental health over and above time spent directly interacting with others by controlling for the latter in the linear model. We found that the number of interaction partners one has positively predicts mental health over and above time spent in these interactions at the daily level, $\beta_{partners} = 4.87$, 95% CI [1.65, 8.08], $t(249) = 2.98$, $p = .003$; $\beta_{hours\ with\ cohabitants} = -0.31$, 95% CI [-2.53, 1.91], $t(249) = -0.27$, $p = .786$; $\beta_{hours\ with\ non\text{-}cohabitants} = 0.46$, 95% CI [-1.90, 2.82], $t(249) = 0.38$, $p = .701$, and marginally at the weekly level, $\beta_{partners} = 2.14$, 95% CI [-0.38, 4.67], $t(249) = 1.67$, p

= .096; $\beta_{\text{hours with cohabitants}}$ = 0.91, 95% CI [-0.51, 2.33], $t(249) = 1.26$, $p = .208$; $\beta_{\text{hours with non-cohabitants}}$ = 0.52, 95% CI [-1.14, 2.19], $t(249) = 0.62$, $p = .536$.

The pandemic has made safe in-person interactions considerably more difficult, resulting in limited opportunities to interact with other people face-to-face on a daily basis. These circumstances may explain why we found no effect of daily in-person interactions on mental health in 2020, when daily in-person interactions were severely limited. Meanwhile, it may have been easier for individuals to find ways to safely connect with other people in-person on a weekly basis, potentially improving mental health via reduced loneliness and increased perceived support, as we saw for virtual interactions. As guidelines began to loosen in early 2021, however, people started meeting in-person more often (Table S2), and we found that both daily and weekly in-person interaction partners were positively associated with mental health. At this timepoint, we found that this association was fully mediated by both decreased loneliness and increased perceived social support. Future research should investigate these findings more thoroughly, and directly compare the effects of the number of in-person versus virtual interaction partners on mental health.
